# Uterine biophysical profile after intrauterine instillation of autologous blood cell derivative (ABCD) for thin endometrium in frozen embryo transfer cycles

**DOI:** 10.1007/s00404-026-08345-y

**Published:** 2026-02-06

**Authors:** Prathyusha Indrakanti, Anjali Mundkur, Vidyashree G. Poojari, Prashanth Adiga, Shivangi Tiwari, Pratap Kumar

**Affiliations:** Department of Reproductive Medicine and Surgery, Kasturba Medical College, Manipal Academy of Higher Education, Manipal, Karnataka 576104 India

**Keywords:** Autologous blood cell derivatives, Uterine biophysical profile, Thin endometrium, Uterine artery PI

## Abstract

**Purpose:**

This study was conducted to investigate the changes in the uterine biophysical profile (UBP) before and after intrauterine administration of Autologous Blood Cell Derivatives (ABCD).

**Methods:**

This prospective observational study investigates endometrial receptivity in frozen embryo transfer (FET) cycles. It focuses on patients with thin endometrium (TEM), a basal serum FSH below 10 IU/L, and good-quality frozen embryos. The study utilizes the UBP to evaluate endometrial receptivity both before and after the ABCD procedure.

**Results:**

The study involved 33 women with TEM undergoing FET cycles. ABCD was administered during the first, second, and third or later FET cycles in 21.21%, 45.45%, and 33.33% of patients, respectively. Following ABCD administration, significant improvements were observed in UBP scores (from 9.06 to 14.85), EMT (from 6.6 mm to 8.67 mm), blood flow to zone III (from 0.85 mm to 3.36 mm), and uterine artery pulsatility index (PI) (from 0.7 to 0.97). Odds ratio analysis showed an association between pregnancy and endometrial layering (OR = 2.12), though it was not statistically significant. Multivariate analysis revealed that the UBP score predicted pregnancy with 68% accuracy, while the ROC analysis yielded an accuracy of 54%. A UBP threshold score of 20 offered 100% specificity, making it a potentially reliable predictor of successful implantation.

**Conclusions:**

This study demonstrates that ABCD growth factors significantly enhance endometrial development in women with TEM, resulting in improved UBP scores, reduced EMT, lower uterine artery PI, and increased uterine blood flow. A UBP score cutoff of 20 demonstrated 95% sensitivity, highlighting its potential as a reliable prognostic tool in infertility treatment.

## What does this study adds to the clinical work

**Table:** 

Using ABCD growth factor infusions is associated with significant improvements in endometrial development, uterine blood flow, and pregnancy outcomes, owing to their standardized, immediate biological action. UBP scoring—particularly a cutoff of 20—shows strong predictive value for successful pregnancy and may serve as a reliable prognostic tool in infertility care.	

## Introduction

Implantation is a complex interplay of dialog between the endometrium and the embryo. Hormonal signals, cellular adhesions, and maternal immune modulation regulate successful embryo implantation. For successful implantation to occur, apposition, adhesion, and invasion must be tightly synchronized with endometrial receptivity, a state influenced mainly by progesterone and estrogen [1].

Thin endometrium (TEM) is seen in 2–3% of infertility treatment cycles. TEM is defined as endometrial thickness (EMT) of < 7 mm in the mid-leuteal phase in frozen embryo transfer cycles [2]. It is one of the common reasons for the cycle cancelation. Various strategies have been employed to mitigate this problem. Increasing the dose of estrogen and the addition of the adjuncts have occasionally failed to improve the EMT. The therapeutic interventions available include pharmacological, regenerative medicine, intrauterine infusion of growth factors, complementary therapies, and autologous platelet-rich plasma.

The uterine biophysical profile (UBP) is an ultrasound-based evaluation of the uterus that examines seven key parameters to assess the uterus’s readiness for implantation and predict the likelihood of a successful pregnancy. These parameters include EMT, layering, uterine artery blood flow as measured by the pulsatility index (PI), myometrial contractions, and echogenicity. UBP includes parameters that determine implantation apart from endometrial thickness evaluation. Factors such as uterine layering, blood flow, uterine myometrial contractions, and a triple line pattern also predict endometrial receptivity. It can be done without any extra equipment or physical training for calculating the parameters. The sum of all the individual parameters determines the UBP score. The UBP can help identify factors affecting endometrial receptivity and predict the likelihood of successful implantation [3]. It is used to assess the uterine environment in patients with unexplained infertility. UBP can be a valuable tool in planning and predicting the outcome of assisted reproductive technologies (ART). A higher UBP score is associated with a higher chance of successful conception.

Autologous Blood Cell Derivatives (ABCD) are next-generation platelet derivatives that increase endometrial thickness. ABCD has shown a promising improvement in the EMT in various studies, also improving pregnancy rates in otherwise canceled cycles [4, 5]. While some aspects have been studied, no research has explored how other uterine factors might shift after ABCD is instilled. Hence, this study aims to investigate changes in the UBP following the intrauterine instillation of ABCD in patients with TEM.

## Materials and methods

This prospective, observational study was conducted at the Department of Reproductive Medicine and Surgery at a tertiary care teaching hospital between April 2023 and September 2024. Ethical approval was obtained from the Institutional Ethics Committee (IEC 1:329/2022), and written informed consent was obtained from participants aged 25–40 years.

The study included patients undergoing FET cycles, excluding patients with endometriosis, endometrial polyps, a history of hematological disorders, premature ovarian failure, and those undergoing donor IVF cycles.

At the beginning of the study, demographic details, including patient age, duration of infertility, and the cause of infertility, were recorded. Additional details were also recorded, including Assisted Reproductive Technology (ART) cycle protocols, UBP parameters, ABCD preparation, embryo transfer (ET), and endometrial parameters before and after ABCD administration, as well as pregnancy outcomes in patients who received ABCD.

### Preparation and administration of ABCD

ABCD growth factor concentrate was prepared in three doses from concentrated platelets obtained from 30 mL of fresh venous peripheral blood once the decision was made. The blood was collected in an EDTA anticoagulant in the proliferative phase and processed to separate discrete components. The upper plasma fraction was separated using a proprietary centrifugation-based selective enrichment method, which prevented disruption of the red blood cell (RBC) layer. With the application of appropriate centrifugal force and time, the procedure is carried out in two phases of centrifugation. The first one separates RBCs and buffy coat from platelet-rich plasma (PRP), and the second one separates platelets in the lower phase with platelet-poor plasma (PPP) in the top phase. The platelet count in whole blood and the platelet-rich fraction was assessed using an automatic blood tester and immunophenotyping with antibodies specific to CD61, CD63, P-selectin, and an early stage platelet activation marker (Beckman Coulter PK7400 Automated Microplate System Analyzer). We transferred the upper fraction of the whole blood into a sterile tube without disrupting the RBC layer, following a proprietary protocol for selective enrichment based on centrifugation. We partitioned a sample volume of 100 µl to determine the concentration and purity of the platelets. After collection, the upper fraction was centrifuged for 12 min. We then transferred the PPP into a tube and obtained platelet particles that were reconstituted. Therefore, platelet concentration was significantly higher, inducing the release of cytokines and growth factors. The next step was centrifugation at 3000 × g for 20 min at 18 °C. We then aliquoted the enriched growth factor concentrate (ABCD) into three 1 mL volumes with PBS. The growth factor concentrate was prepared from approximately 600 × 10^6 platelets in the final concentrate.

### Administration protocol

Three ABCD growth factor concentrate doses were prepared from 30 ml of peripheral blood. The first dose was administered intrauterinely using an insemination catheter when a thin endometrium was diagnosed after 7–10 days of endometrial preparation. The second and third doses were stored frozen at − 18 to − 26 ^°^C for later use. The second dose was administered 2–3 days after the first, and the third dose was instilled on the day progesterone treatment began. Hormone replacement therapy (HRT) frozen embryo transfer cycles with a maximum dose of estradiol valerate 12 mg per day, which failed to show an improvement in endometrial thickness of at least 7 mm, were recruited. The UBP score was recorded both before and after three doses of ABCD. Progesterone vaginal gel was started on days 14, 15, or 16, and embryo transfer was performed according to the day of the embryo, either on day 3 or 5. Only grade 1 or 2 embryos were used for the transfer, which were fertilized by Intracytoplasmic sperm injection (ICSI).

A total of 33 patients were recruited to the study, assuming a standard deviation of Endometrium thickness of 0.85 mm and a clinically significant difference of 0.5 mm [3] at a 5% level of significance and 90% power.

The collected data were entered into Microsoft Excel and analyzed using the statistical software GraphPad InStat v3.0. Quantitative data were summarized as Mean and Standard Deviation for normally distributed variables, or as Median (Q1, Q3) for variables that were not normally distributed. Qualitative data were expressed as frequencies and percentages. A paired *t* test was used to assess changes in variables before and after ABCD treatment for normally distributed data. At the same time, the Wilcoxon signed-rank test was used for non-normally distributed variables. For categorical variables, the McNemar test was employed. A *p* value of less than 0.05 was considered statistically significant.

## Results

The study involved 33 women diagnosed with TEM undergoing FET cycles. The mean age of the participants was 33.97 ± 4.23 years, with an average duration of infertility of 7.36 ± 3.74 years. The height and weight of each participant were recorded to calculate their body mass index (BMI), which averaged 24.5 ± 4.04 kg/m^2^. Table 1 provides an overview of the demographic characteristics of the participants.

**Table 1:** Demographic details of patients (*N* = 33)

Demographic details	Description	Frequency	Percentage
Age distribution	20–24 years	1	3.03
	25–29 years	3	9.09
	30–34 years	14	42.42
	35–39	11	33.33
	40–44 years	4	12.12
Type of infertility	Primary	14	42.42
	Secondary	19	57.58
Cause of infertility	Female	12	36.36
	Male	6	18.18
	Combined	7	21.21
	Unexplained	8	24.24
Menstrual history	Regular	30	90.91
	Irregular	3	9.09

It was found that 12 patients (36.36%) had a history of hypothyroidism, while one patient each (3.03%) had a history of pulmonary tuberculosis and anemia with hypertension, respectively. Fourteen patients (42.42%) had previously undergone surgery for various indications, whereas 19 patients (57.58%) reported no history of prior surgeries.

ABCD was administered during the first FET cycle in 7 patients (21.21%), in the second FET cycle for 15 patients (45.45%), and during the third or later cycles in 11 patients (33.33%). The embryo transfer procedure was complex, with a duration of catheter insertion exceeding 1 min for five patients (15.15%), but was easily performed in the remaining 28 patients (84.85%).

The primary objective was to measure the uterine biophysical profile scoring before and after administration of ABCD. Statistical analysis revealed a significant increase in UBP total scores (from 9.06 ± 2.87 pre-ABCD to 14.85 ± 3.28 post-ABCD), endometrial thickness (from 6.6 ± 0.44 mm pre-ABCD to 8.67 ± 1.14 mm post-ABCD blood flow to zone III (from 0.85 ± 1.7 mm pre-ABCD to 3.36 ± 2.04 mm post-ABCD), and mean PI of the uterine artery (from 0.7 ± 0.81 pre-ABCD to 0.97 ± 0.85 post-ABCD), all with a *p* value of 0.0001 based on paired *t* test analysis.

While an increasing trend was observed in variables such as myometrial contraction, endometrial layering, ET scores, mean PI of the uterine artery, and blood flow to the myometrium, these changes were not statistically significant (*p* > 0.05). A detailed comparison between pre- and post-ABCD parameters is summarized in Table 2.

**Table 2:** Comparison of UBP variables pre- and post-ABCD in 33 patients. *Represents *p* < 0.05 on paired *t* test

UBP Variable	Pre-ABCD		Post-ABCD		Paired *t* test *t* value	*p* value
	Mean (mm)	SD	Mean (mm)	SD		
Total score—UBP	9.06	2.87	14.85	3.28	9.29	0.00001*
EMT	6.60	0.44	8.67	1.14	11.99	0.0001*
Myometrial contraction	2.36	1.25	2.64	0.99	1.788	0.08
EMT score	0.00	0.00	2.30	0.64	NA	NA
Endometrial layering	1.48	1.23	1.79	1.17	1.71	0.09
Mean PI of uterine artery	0.70	0.81	0.97	0.85	2.05	0.04*
Blood flow zone III	0.85	1.70	3.36	2.04	7.05	0.0001*
Blood flow myometrial	2	0	2	0	NA	NA

*ABCD* Autologous Blood Cell derivative; *EMT* endometrial thickness, *PI* pulsatility index; *UBP* uterine biophysical profile

**Fig. 1:**
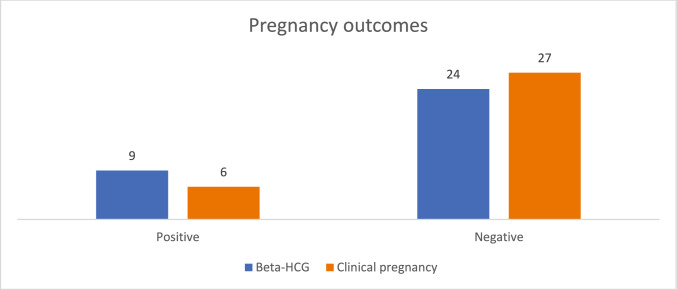
Pregnancy outcomes in 33 patients

A total of 33 patients were prospectively monitored to evaluate pregnancy outcomes, including biochemical and clinical pregnancy rates. Figure 1 depicts the pregnancy rates observed during the study. Among the participants, four patients (12.12%) achieved live births, and one patient (3.03%) was confirmed to be pregnant following treatment. However, pregnancy was not detected in the remaining 27 patients (84.85%).

Later in the study, we analyzed the correlation between beta-HCG levels and EMT. Our findings showed that six patients with an EMT increase of 0 to 1.9 mm had positive beta-HCG results. Similarly, one patient with an EMT increase between 2 and 2.9 mm also tested positive for beta-HCG. In contrast, two patients showed positive beta-HCG levels when their EMT increased by 3 mm or more.

**Fig. 2:**
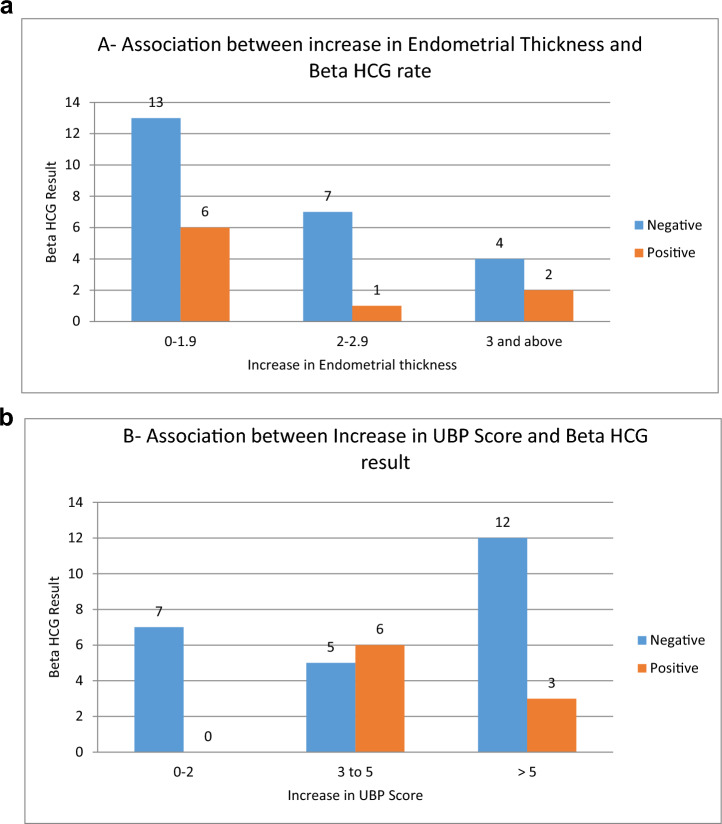
Association between an increase in (**A)** endometrial Thickness and Beta-HCG positive, (**B)** UBP Score and Beta-HCG positive

We examined the relationship between an increase in UBP scores and beta-HCG levels. It was observed that patients with a UBP score increase of 0–2 did not show positive beta-HCG results. Conversely, six patients had positive beta-HCG when their UBP score increased by 3–5, and three patients registered positive beta-HCG levels with a UBP score increase of more than 5, refer to Fig. 2A, B for a visual summary.

The odds ratio (OR), a statistical measure of the relationship between an exposure and an outcome, was calculated to assess the association between positive pregnancy outcomes and UBP scores. The *p* value is highly dependent on the sample size. Therefore, even a strong OR may not reach statistical significance. The analysis revealed higher odds of positive pregnancy outcomes in the following parameters:

- Increased endometrial thickness (OR = 1.040, 95% CI 0.392–2.75, *p* = 0.941)
- Enhanced endometrial layering (OR = 2.120, 95% CI 0.72–6.23, *p* = 0.171)
- Elevated ET scores (OR = 1.070, 95% CI 0.171–6.72, *p* = 0.941)
- Higher mean PI values (OR = 1.66, 95% CI 0.592–4.67, *p* = 0.332)

Among these, endometrial layering demonstrated the strongest association with pregnancy rates, with an OR of 2.12, suggesting that an OR > 1 correlates with increased likelihood of positive pregnancy outcomes. However, other UBP score variables showed no significant association, as their OR values were below 1. Table 3 provides a detailed summary of these findings.

**Table 3:** Odds of UBP score and positive pregnancy outcome

Odds of UBP score and positive pregnancy outcome	Odds ratio	Lower 95%CI	Upper 95%CI	*p* value
(Intercept)	0.447	841.00	841.00	0.834
EMT	1.040	0.392000	2.75	0.941
Endometrial Blood flow	0.994	0.638000	1.55	0.980
Endometrial Layering	2.120	0.723000	6.23	0.171
EMT score	1.070	0.171000	6.72	0.941
Myometrial Echogenicity	0.254	0.015100	4.27	0.341
Myometrial Contractions	0.946	0.353000	2.54	0.913
PI	1.660	0.594000	4.67	0.332

*EMT* endometrial thickness, *PI* pulsatility index; *UBP* uterine biophysical profile

A multivariate regression analysis (MLR) model yielded an area under the curve (AUC) of 68%, indicating that the combined parameters of the UBP score could predict successful pregnancies in 68% of cases. However, the UBP score’s receiver operating characteristic (ROC) analysis yielded an AUC of 54%, which fell short of the ideal score of 20 due to the method’s focus on maximizing sensitivity and specificity.

**Fig. 3:**
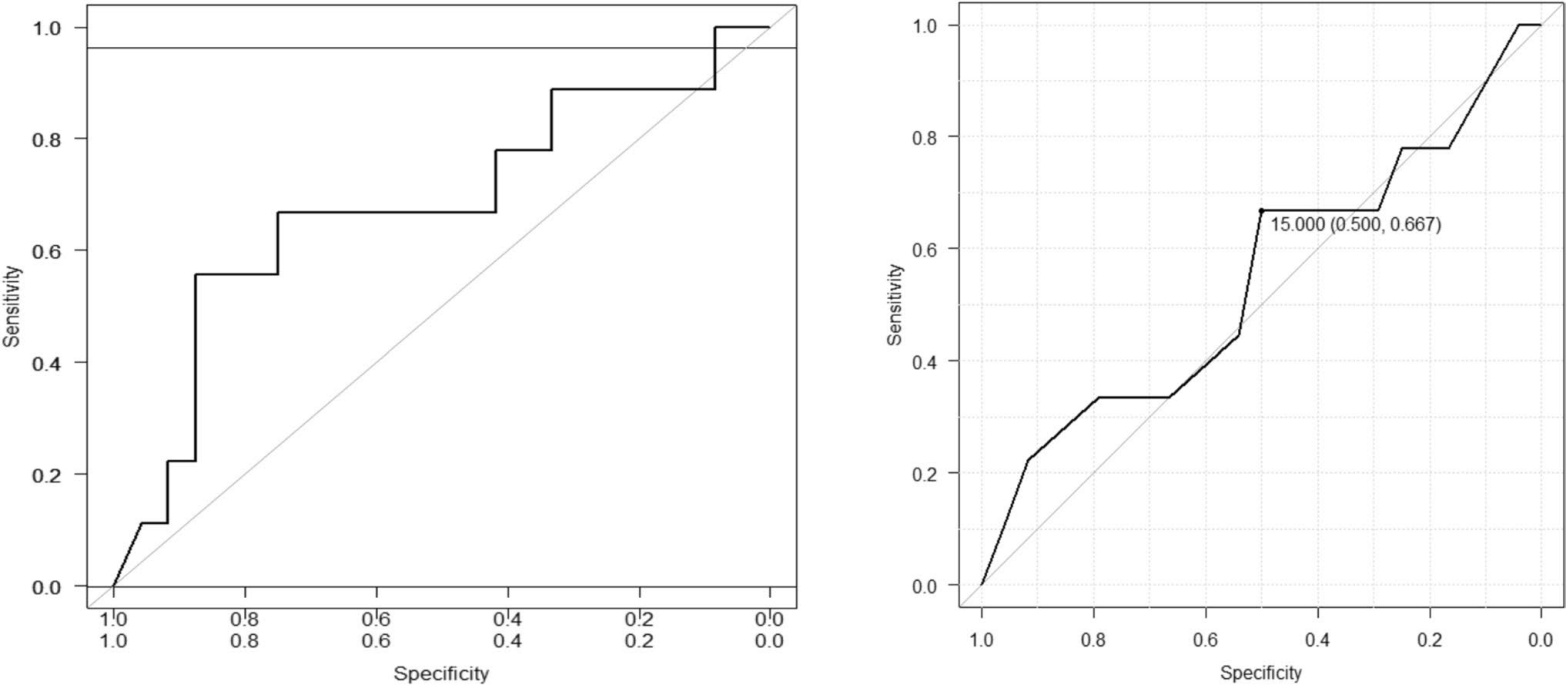
(**A)** Multivariate regression analysis model fitted MLR analysis; (**B)** ROC of UBP total scores in predicting positive pregnancy outcomes

To effectively use the UBP score as a predictive tool, a threshold of 20 appears optimal, offering 100% specificity. This aligns well with another research that has validated the UBP score. While a cutoff of 15 yielded 50% specificity, raising it to 20 significantly boosted specificity to 95%. Figure 3A, B illustrates the multivariate regression model and the ROC analysis for UBP parameters.

## Discussion

Several studies have shown improvement in endometrial thickness following the administration of ABCD [5, 6]. ABCD is a growth factor-rich concentrate compared to platelet-rich plasma [1]. UBP, as described by Malhotra et al., was used in this study. A seven-parameter scoring system with a maximum score of 20 points was applied. We used the same scoring system before and after ABCD instillation to evaluate changes and improvements in the uterine biophysical profile. The most notable changes in UBP parameters were observed in the total UBP score, EMT, blood flow to Zone III, and the mean PI of the uterine artery. These changes suggest significant alterations in uterine blood flow following ABCD instillation. Interestingly, this study uncovered important insights from patients with lower scores, thereby expanding on the original scoring system’s primary purpose of predicting successful pregnancy outcomes.

This study demonstrated a significant increase in EMT following treatment, although a corresponding rise in pregnancy rates did not accompany this. These findings are consistent with those of Tiwari S et al., who reported significant improvements in EMT after intrauterine ABCD infusions—specifically, mean increases of 1.36 mm in the TEM group and 0.77 mm in the normal endometrium group, both of which were statistically significant [6]. Similarly, Vasanthi P et al. found that 98% of patients experienced substantial endometrial thickening, with optimal responses (EMT > 7 mm) observed after the second dose of ABCD [7].

Among the UBP parameters, greater EMT, improved endometrial layering, higher ET scores, and elevated mean PI were associated with favorable pregnancy outcomes. Endometrial layering showed the strongest correlation (odds ratio = 2.12) with favorable pregnancy outcomes. The study also found a positive relationship between higher total UBP scores and increased pregnancy rates.

Receiver operating characteristic (ROC) analysis revealed a specificity of 50% at a UBP score cutoff of 15, which increased to 95% at the ideal score of 20. These findings align with those of Sedeq MG et al., who reported a sensitivity of 86.7% and a specificity of 100% at a UBP cutoff of 15 [8]. Key predictors of pregnancy in their study included endometrial thickness between 10 and 14 mm, well-defined trilaminar endometrial layers, and robust blood flow in Zone III.

Key factors significantly associated with higher pregnancy rates included an EMT of 10–14 mm (*p* < 0.001), a prominent trilaminar endometrial pattern (*p* < 0.001), abundant blood flow in Zone III (*p* < 0.001), and a uterine artery Doppler PI of less than 2.19 (*p* < 0.001).

The broader inclusion criteria used in their study likely contributed to the higher sensitivity, enabling the identification of a broader range of predictive factors relevant to successful pregnancy outcomes. These findings underscore the utility of UBP as a reliable predictive tool in infertility management, highlighting the importance of comprehensive uterine assessment in optimizing reproductive outcomes.

This study provides early evidence on using ABCD growth factors in women with thin endometrium undergoing ART. What makes this research particularly innovative is the inclusion of uterine morphological parameters, specifically the UBP scoring system. With a larger sample size and multicenter randomized controlled trials, UBP may emerge as a solution for endometrial receptivity and may outperform individual parameters. The method of assessing UBP score is simple, quick, and more reliable, as the parameters studied have shown statistical significance individually in terms of pregnancy outcomes. UBP score assessment can be performed routinely in clinical practice.

However, as a small, prospective, self-controlled study without a randomized control group, it is subject to inherent limitations. These include potential bias due to patient selection preferences during enrolment and the constraints imposed by the limited sample size. To validate these findings, further research is essential, particularly studies aimed at elucidating the underlying biological mechanisms and involving larger cohorts within well-designed randomized controlled trials. Significantly, no clinical research has directly compared platelet-rich plasma (PRP) with ABCD growth factors in infertility patients concerning changes in the uterine milieu. This gap presents a valuable opportunity for future investigations to explore and expand the therapeutic potential and comparative efficacy of these innovative treatments. Patient characteristics, such as increased age, baseline platelet count and function, hormonal milieu, metabolic and nutritional status, can be evaluated in future research.

## Conclusions

Our research indicates that ABCD growth factors are more effective in fostering endometrial development than PRP alone, as the presence of growth factors is known to be in standardized concentrations and acts immediately on the endometrial cells. Growth factors from PRP depend on release, platelet count, activation method, and patient characteristics. Women who previously struggled with TEM saw considerable improvements in their uterine morphokinetic parameters after receiving ABCD infusions. This included an increase in total UBP scores, greater EMT, a healthier uterine artery PI, and stronger blood flow to Zone III. Post-treatment outcomes were encouraging, with approximately 27% of patients achieving a positive β-hCG, 18% having confirmed clinical pregnancies, and a 12% live birth rate observed. Our findings indicated a strong correlation between successful pregnancy outcomes and several key parameters, including endometrial thickness, endometrial layering, ET score, and uterine artery PI. Notably, a UBP score cutoff of 20 demonstrated 95% sensitivity in predicting favorable pregnancy outcomes, indicating that UBP scoring holds considerable promise as a reliable screening and prognostic tool in infertility treatment.

## Data Availability

De-identified patient data used for this study is available upon request.
